# Beneficial Effects of Non-Encapsulated or Encapsulated Probiotic Supplementation on Microbiota Composition, Intestinal Barrier Functions, Inflammatory Profiles, and Glucose Tolerance in High Fat Fed Rats

**DOI:** 10.3390/nu11091975

**Published:** 2019-08-22

**Authors:** Sunhye Lee, Rebecca Kirkland, Zachary I. Grunewald, Qingshen Sun, Louise Wicker, Claire B. de La Serre

**Affiliations:** 1 Department of Anatomy, Physiology, and Cell Biology, University of California Davis School of Veterinary Medicine, Davis, CA 95616, USA; 2 Department of Foods and Nutrition, University of Georgia, Athens, GA 30602, USA; 3 Department of Nutrition and Exercise Physiology, University of Missouri, Columbia, MO 65211, USA; 4 College of Life Science, Heilongjiang University, Harbin 150080, China; 5 School of Nutrition and Food Sciences, Louisiana State University AgCenter, 101 LSU Union Square, Baton Rouge, LA 70803, USA

**Keywords:** probiotics, microencapsulation, gut microbiota, intestinal epithelial barrier, inflammation, glucose tolerance

## Abstract

Development of obesity-associated comorbidities is related to chronic inflammation, which has been linked to gut microbiota dysbiosis. Thus, modulating gut microbiota composition could have positive effects for metabolic disorders, supporting the use of probiotics as potential therapeutics in vivo, which may be enhanced by a microencapsulation technique. Here we investigated the effects of non-encapsulated or pectin-encapsulated probiotic supplementation (*Lactobacillus paracasei subsp. paracasei* L. casei W8^®^; L. casei W8) on gut microbiota composition and metabolic profile in high-fat (HF) diet-fed rats. Four male Wistar rat groups (*n* = 8/group) were fed 10% low-fat, 45% HF, or HF with non-encapsulated or encapsulated L. casei W8 (4 × 10^7^ CFU/g diet) diet for seven weeks. Microbiota composition, intestinal integrity, inflammatory profiles, and glucose tolerance were assessed. Non-encapsulated and pectin-encapsulated probiotic supplementation positively modulated gut microbiota composition in HF-fed male rats. These changes were associated with improvements in gut barrier functions and local and systemic inflammation by non-encapsulated probiotics and improvement in glucose tolerance by encapsulated probiotic treatment. Thus, these findings suggest the potential of using oral non-encapsulated or encapsulated probiotic supplementation to ameliorate obesity-associated metabolic abnormalities.

## 1. Introduction

Obesity and its associated comorbidities are major health problems in Western countries, particularly the United States where energy-dense diets are commonly consumed [1]. Obesity is a state of chronic systemic low-grade inflammation [2]. Visceral adiposity, especially mesenteric, is a strong predictor of systemic inflammation, insulin resistance and other metabolic dysfunctions [2,3].

Inflammation in obesity has been shown to originate, at least in part, from the gastrointestinal (GI) tract [4].We have previously found propensity to high-fat (HF) diet-induced obesity is associated with an increase in GI permeability [4]. Chronic HF feeding impairs GI barrier integrity, particularly in the distal gut (ileum, colon), notably by altering the expression of gut-protecting mucins [5]. The distal gut harbors more than 10^14^ microbial organisms [6], and increases in gut permeability in combination with diet-driven alteration of gut microbiota composition (dysbiosis) allows for potential passage of pro-inflammatory bacterial products, such as lipopolysaccharide (LPS) to the circulation, promoting systemic inflammation [7,8]. Interestingly, chronic administration of LPS in mice and rats has been shown to induce excessive weight gain, adipose tissue inflammation and hepatic insulin resistance [7,9], highlighting a potential causal role for microbiota-driven inflammation in metabolic disorders. Additionally, normalization of diet-driven dysbiosis restores gut epithelial barrier and improves metabolic outcomes, especially glucose homeostasis [10].

Probiotics are defined by the FAO/WHO as live microorganisms which, when administered in adequate amounts, confer a health benefit on the host [11]. Probiotics exert their beneficial effects via positive modulation of the host gut microbiota composition. They can inhibit the growth of pathogenic bacteria such as *Clostridium perfringens* and/or promote the proliferation of beneficial populations such as *Bifidobacteria* [12]. Probiotics inhibits the growth of enteric pathogens via the production of inhibitory antimicrobials, competitively adhering to the mucosa and epithelium, strengthening the epithelial barrier integrity via mucin and defensins, and modulating the immune system of the host [13]. Furthermore, probiotic-driven changes in gut microbiota can lead to changes in the production of intestinal short chain fatty acids (SCFAs) [14], promoting the production of GI peptides, including glucagon-like peptide-1 (GLP-1) [15]. GLP-1 is secreted by enteroendocrine L cells in the distal gut in response to feeding and acts as an incretin [16]. The therapeutic effects of probiotics on metabolic diseases have been implicated in many experimental and clinical studies [17], and probiotics can alleviate various aspects of metabolic disorders. A variety of beneficial strains, especially *Lactobacillus*, *Streptococcus* and *Bifidobacterium*, have been shown to positively affect hyperlipidemia, obesity and insulin resistance in HF diet-fed animals in association with changes in gut microbiota composition and maintenance of gut epithelial integrity [18].

In order to exert their functions, high concentration of viable probiotic bacteria have to be able to reach the distal part of the intestine where they can interact with commensal microbiota. Probiotics are exposed to environmental conditions during processing and production of foods that decrease cell number and viability, including exposure to oxygen, heat, humidity, light, and shear that damage the cell wall, cell membrane or result in damage related to oxidation [19]. Furthermore, the delivery of orally ingested probiotics may be restricted by the harsh environmental conditions of the GI tract (e.g., gastric acid, digestive enzymes, and bile salts of the small intestine); thus, it may be desirable to develop methods that increase the viability of probiotic cells until they reach the lower GI tract. 

In this regard, the recent emergence of the microencapsulation technique appears to be a potential solution to enhance the survivability of probiotics [20]. Encapsulation technologies include physical methods and biopolymer entrapment, which are highly effective for targeted delivery of bioactives in functional foods [21] and often applied to probiotics [22]. A carbohydrate-based biopolymer, pectin, is a soluble fiber that survives transit through the GI system prior to fermentation by colonic bacteria and is frequently used as a bio-carrier for drugs [23]. Ionotropic gelation and entrapment of bioactives are attributed to the charged homogalacturonan domain of pectin [23]. De-esterification of pectin by plant pectinmethylesterase compared to fungal pectinmethylesterase or chemical saponification results in highly reactive blocks of charge distribution at the same total charge and higher gel strength [24,25] and lower release rates of a model drug indomethacin [26]. *Lactobaccillus casei* encapsulated by block de-esterified pectin showed greater survival during storage and greater stability under simulated GI transit [27].

The present study investigated the potential beneficial effects of probiotic and microencapsulated probiotic bacteria, *Lactobacillus paracasei subsp. Paracasei* L. casei W8^®^ (L. casei W8), in the prevention of HF diet-induced metabolic disorders. We hypothesized that probiotic supplementation would improve diet-induced inflammation and epithelial barrier integrity associated with positive alterations in intestinal microbiota composition. We anticipated the microencapsulation would significantly enhance delivery and effects of the probiotic. To test this hypothesis, Wistar rats were fed an HF diet supplemented with a non-encapsulated or pectin-encapsulated probiotic for seven weeks and gut microbiota composition, inflammation and glucose tolerance were determined.

## 2. Materials and Methods

### 2.1. Preparation of Microencapsulated L. casei

The lactic acid bacterial strain, L. casei W8, was donated by Chr. Hanson, Hørsholm, Denmark and stored at −80 °C until use. Pectin used for charge modification and encapsulation of cells was GENU pectin type B rapid set-Z from citrus peel, DE 72%, Batch NO: GR41649, and was donated by CP Kelco, Copenhagen, Denmark. Pectinmethylesterase was extracted from Valencia citrus pulp (donated by Citrus World, Lake Wales, FL, USA). Charge modification and encapsulation of *L. casei* cells was completed as described [27], similarly to the method used to encapsulate indomethacin [26]. Briefly, pectin degree of esterification was decreased from 72% to 35% by the addition of known units of pectinmethylesterase and catalysis ensued for a period of time to remove the desired amount of methoxyl ester. Charge modified pectin was hydrated in water, mixed with *L. casei* cells, hydrated in peptone water to a target of log cfu/mL of 12, and encapsulation by iontropic gelation was achieved by extrusion into 300 mM calcium chloride. Recovered beads were freeze-dried until use. Viable flora was enumerated by plate counting method on MRS agar at 37 °C [28]. Encapsulated and non-encapsulated beads were blended with HF (45% kcal as fat, Research Diets, New Brunswick, NJ, USA) diet at 4 °C using a bowl chopper (Schneidmischer, Kramer and Grebe KG., Ludwig, DE, USA) to achieve a final concentration of 4 × 10^7^ CFU/g. The blended diets were stored at −20 °C throughout the study.

### 2.2. Animals and Treatment Regimen

Thirty-two male Wistar rats (200~220 g, Envigo, Indianapolis, IN) were single-housed in a temperature-controlled room with a 12-h light-dark cycle. Animals were divided into four groups (*n* = 8 per group) and fed low fat (LF; 10% kcal as fat, Research Diets #12450H), HF (45% kcal as fat, Research Diets #12451), HF supplemented with non-encapsulated L. casei W8 (HF/Pro; 4 × 10^7^ CFU/g diet), or HF with supplemented with encapsulated L. casei W8 (HF/Pca; 4 × 10^7^ CFU/g diet) for seven weeks (Tables S1 and S2). Bodyweight and food intake were monitored daily. Food intake (g) was determined by subtracting the number of remaining diets in the cages on day *n* (including food at the bottom of the cages and crumbs) from the amount provided on day (*n*-1). Energy intake (Kcal) was then calculated using the caloric density of each diet (3.82 kcal/g diet for LF and 4.7 kcal/g diet for HF groups; Table S2).

After seven weeks on their respective diets, animals were fasted for 6 h and euthanized via CO_2_ inhalation. For all animals, euthanasia took place within 3 hours after the beginning of the light cycle and sacrifice order was randomized among groups over 4 days. Serum was obtained from blood collected by cardiac puncture, rested on ice for 15 min, and spun (3000 rpm for 10 min at 4 °C). Liver, ileum, cecum, colon, and visceral fat pads (mesenteric, retroperitoneal, and epididymal) were dissected, weighed, and an adiposity index was determined. Serum and all tissues were snap-frozen and stored at −80 °C until analysis. All animal care procedures were approved by the Institutional Animal Care and Use Committee of the University of Georgia (approval number: A2013 09-002-Y2-A5).

### 2.3. Oral Glucose Tolerance Test (OGTT)

After six weeks on their respective diets, animals were fasted for 5 h before oral gavage with a glucose solution (2 g/kg bodyweight using 20% glucose, Sigma-Aldrich, St. Louis, MO, USA). Glycemia was measured using a glucometer (Freestyle, Alameda, CA, USA) before (0 min) and after (15, 30, 60, 90, and 120 min) glucose challenge. Blood samples (~100 μL) were collected by tail bleeding (tail snip) at each time point to determine circulating insulin levels.

### 2.4. Microbiota DNA Sequencing

DNA was extracted from cecal contents (ZR Fecal DNA MiniPrep, Zymo Research, Irvine, CA) and the eluted DNA was sent to SeqMatic LLC (Fremont, CA) for PCR, sequence library preparation, and sequencing. Briefly, polymerase chain reaction (PCR) was used to target the V4 region of the 16S rRNA genes using the 515F/806R primer set based on the protocol used by the Earth Microbiome project [29]. Sequence reads were obtained from a paired end 2 × 150 Illumina MiSeq run. Sequences were then matched to reference sequences for taxonomic determination. Bacterial abundances were determined and all phylogenic level and normalized by log transformation. The METAGENassist platform [30] was used for multivariate statistical analysis. The Galaxy platform was used to perform linear discriminant analysis effect size on log-transformed abundance and identify representative taxa for each group [31]. Differences in abundances among groups were assessed using one-way ANOVA with Kruskal–Wallis test with Dunn’s post hoc test.

### 2.5. SCFA Analysis

Serum samples were used for SCFAs quantification, which was done at the Mayo Clinic Metabolomics Core using gas chromatography–mass spectrometry as previously described [32].

### 2.6. Biochemical Analyses

Insulin (Alpco, Salem, NH, USA) and LPS-binding protein (LBP, proxy for LPS levels; Biometec, Greifswald, Germany) in serum were measured as a via enzyme-linked immunosorbent assay (ELISA) according to manufacturers’ instructions.

### 2.7. Real-Time PCR (qPCR)

mRNA was extracted from distal gut (ileum) and visceral (mesenteric) fat tissues using the RNeasy Mini Kit or Lipid Tissue Mini Kit (Qiagen, Valencia, CA) to determine expression for inflammatory genes. Ileum tissues were used to assess gene expression of epithelial barrier function markers. Following extraction, mRNA quantity and purity was determined using a NanoDrop ND-1000 spectrophotometer (NanoDrop Technologies, Wilmington, DE, USA). and complementary DNAs (cDNAs) were synthetized by reverse transcription (RevertAid™ First Strand cDNA Synthesis Kit, Thermo Fisher Scientific, Franklin, MA). cDNAs were then amplified by qPCR (StepOnePlus real-time PCR, Thermo Fisher Scientific) using SYBR Green PCR master mix (Thermo Fisher Scientific) and primers purchased from Integrated DNA Technologies (Table S3). Gene expression levels were determined using the 2^−ΔΔCt^ method [33].

### 2.8. Statistical Analysis

Data are presented as mean ± SEM. Unless noted otherwise (microbiota analysis), Prism software (Prism 6.0; GraphPad Software, La Jolla, CA) was used for statistical analysis. Bodyweight, energy intake, and OGTT were analyzed by two-way repeated measures ANOVA while one-way ANOVA was used to analyze adiposity levels, gene expression data, SCFAs levels, and biochemical analyses. Differences were considered significant if *p* < 0.05.

## 3. Results

### 3.1. Effects of Probiotic Administration on Bodyweight, Energy Intake, and Adiposity

**Figure 1:**
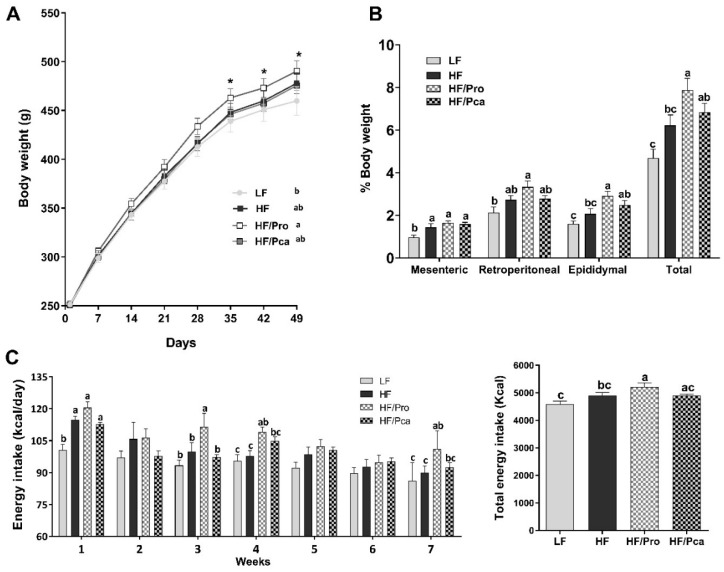
Non-encapsulated or encapsulated probiotic supplementation had no protective effects on obesity phenotype. Bodyweight (**A**), adiposity index: Fat pad weight/BW (%) (**B**) and energy intake (**C**) of rats fed an LF, HF, HF/Pro, or HF/Pca diet for seven weeks. Values are means ± SEMs; *n* = 8/group. ^a,b,c^ means with different letters are statistically significant, * indicates *p* < 0.05. HF, high fat; HF, high fat; HF/Pca, HF with supplemented with encapsulated *L. casei* W8 (4 × 10^7^ cfu/g diet); HF/Pro, HF supplemented with non-encapsulated *L. casei* W8 (4 × 10^7^ cfu/g diet); LF, low fat.

There were significant differences in bodyweight at baseline among all treatment groups (Figure 1A). After seven weeks on respective diets, all HF fed groups showed higher bodyweight than the control LF group, but this difference only reached significance in the probiotics-supplemented animals (LF vs. HF/Pro, *p* < 0.05). The HF and HF/Pca groups’ bodyweight was not statistically different from either the LF or HF/Pro group (*p* > 0.05).

Despite minimal differences in bodyweight, there were differences in adiposity among groups. Generally, probiotics-treated animals exhibited greater adiposity than the LF control group, particularly the naked probiotic rats. Similarly to bodyweight, the HF/Pro group had a significantly higher total adiposity index than LF and HF rats (HF/Pro vs. LF, *p* < 0.0001; vs. HF, *p* < 0.05; Figure 1B), but not than HF/Pca rats, which was mainly driven by the differences in retroperitoneal fat pad weights (HF/Pro vs. LF, *p* < 0.01; vs. HF, *p* = 0.08) and epididymal fat pad weights (HF/Pro vs. LF, *p* < 0.001; vs. HF, *p* < 0.01) fat pads. All three HF fed groups showed a significant increase in mesenteric fat depot compared to the LF group (*p* < 0.01).

There were no significant differences in total energy intake among LF, HF and HF/Pca rats throughout the experiment. The HF/Pro rats consumed significantly more overall than LF and HF rats (HF/Pro vs. LF, *p* < 0.001; vs. HF, *p* < 0.05), but not more than the HF/Pca groups (*p* = 0.056).

During the 1st week, HF-fed groups had a significantly higher energy intake than the LF group (LF vs. HF, *p* < 0.05; vs. HF/Pro, *p* < 0.001; vs. HF/Pca, *p* < 0.05; Figure 1C). Energy intake was normalized throughout the rest of the experiment except for the Pro group which consumed significantly more calories than the LF and HF fed rats on week 3, 4 and 7 (week 3: HF/Pro vs. LF, *p* < 0.01, vs. HF, *p* < 0.05, vs. HF/Pca, *p* < 0.01; week 4: HF/Pro vs. LF, *p* < 0.05, vs. HF, *p* < 0.05, vs. HF/Pca, *p* = ns; week 7: HF/Pro vs. LF, *p* < 0.01, vs. HF, *p* < 0.05, vs. HF/Pca, ns).

### 3.2. Effects of Probiotic Administration on Glucose Homeostasis

**Figure 2:**
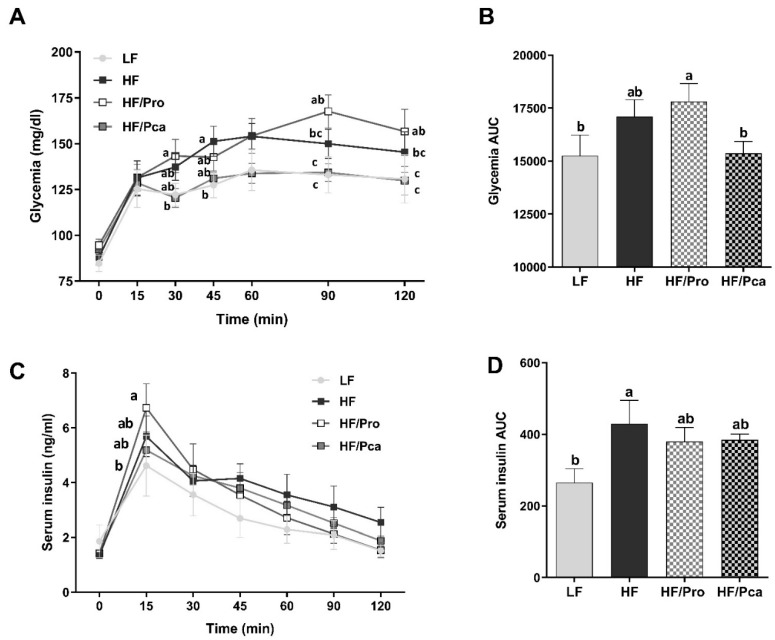
Encapsulation of probiotic bacteria improved glucose intolerance. Glycemia (**A**,**B**) and insulinemia (**C**,**D**) were measured during an OGTT (2 mg/kg) in rats fed an LF, HF, HF/Pro, or HF/Pca diet for seven weeks. Values are means ± SEMs; *n* = 8/group. ^a,b,c^ means with different letters are statistically significant, *p* < 0.05. HF, high fat; HF/Pca, HF with supplemented with encapsulated *L. casei* W8 (4 × 10^7^ cfu/g diet); HF/Pro, HF supplemented with non-encapsulated *L. casei* W8 (4 × 10^7^ cfu/g diet); LF, low fat.

There were no differences in baseline glucose and insulin levels among groups (Figure 2A,C). Following an OGTT, glycemia constantly increased for 60 min (LF, HF, and HF/Pca) or 90 min (HF/Pro) post challenge. HF alone resulted in a non-significant constant increase in glycemia following the glucose challenge. Similarly, HF/Pro rats displayed higher glycemia throughout the 2-hr experiment. HF/Pro rats’ glycemia was significantly higher than the LF control rats at 90 and 120 min (HF/Pro vs. LF, *p* < 0.01) and their overall glycemia area under the curve (AUC) over 120 min was also significantly higher compared to LF rats (HF/Pro vs. LF, *p* < 0.001), but not to the HF group (Figure 2B). The HF/Pca fed rats’ response to the OGTT challenge was similar to the LF rats. HF/Pro glycemia levels were significantly lower than HF/Pca rats’ levels at the same 90 and 120 min time point (HF/Pca vs. HF/Pro, *p* < 0.01) and their overall AUC was also significantly lower (HF/Pca vs. HF/Pro, *p* < 0.001).

Serum insulin levels peaked at 15 min post oral glucose challenge in all groups with a significant elevation in the HF/Pro group compared to the LF group (*p* < 0.05), but not to HF and HF/Pca rats (Figure 2C). HF feeding significantly increased serum insulin AUC (LF vs. HF, *p* < 0.05), which was reduced by probiotic treatment, albeit not significantly (Figure 2D).

### 3.3. Effects of Probiotic Administration on Gut Microbiota and Metabolites

**Figure 3:**
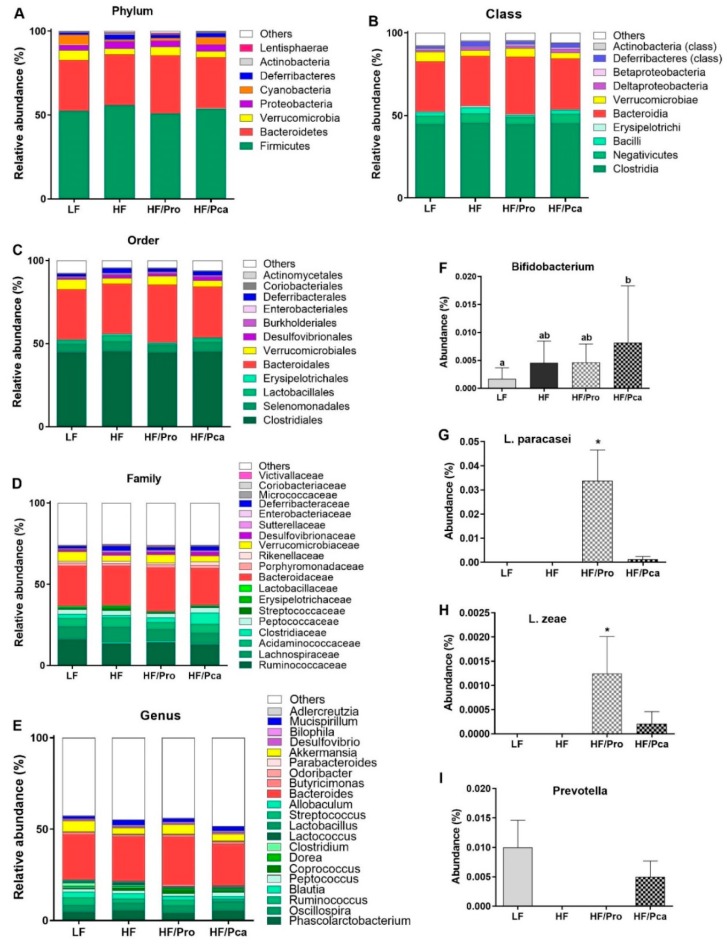
Non-encapsulated or encapsulated probiotic supplementation led to compositional changes in gut microbiota. Microbial composition at the phylum (**A**), class (**B**), order (**C**), family (**D**) genus I, and species (**F**–**I**) of rats fed an LF, HF, HF/Pro, or HF/Pca diet for seven weeks. *n* = 8/group. Asterisk (*) or ^a,b,c^ means with different letters are statistically significant, *p* < 0.05. All phylogenic levels present with abundance >1% are represented. HF, high fat; HF/Pca, HF with supplemented with encapsulated *L. casei* W8 (4 × 10^7^ cfu/g diet); HF/Pro, HF supplemented with non-encapsulated *L. casei* W8 (4 × 10^7^ cfu/g diet); LF, low fat.

Gut microbiota was analyzed at all phylogenic levels with an abundance > 1% represented (Figure 3A). HF consumption altered the microbiota composition. Especially, at the phylum level (Figure 3A), the HF groups had a significantly higher ratio of the main phyla *Firmicutes* to *Bacteroidetes* than the LF group (*p* < 0.05), which was normalized by probiotic supplementation in the HF/Pro group (HF/Pro vs. HF, *p* < 0.05), and partially improved by the HF/Pca treatment. Furthermore, HF feeding alone led to significant increases in abundance of *Deltaproteobacteria* (*p* < 0.05) and *Actinobacteria* (*p* < 0.05), mediated by increases in *Coriobacteriales* (order, *p* < 0.001), *Coriobacteriaceae* (family, *p* < 0.001), *Adlercreutzia* (genus, *p* < 0.05) and *Desulfovibrionales* (order, *p* < 0.05), *Desulfovibrionaceae* (family, *p* < 0.05), *Bilophila* (genus, *p* < 0.001), respectively (Figure 3A–E, Figure S1).

In LF-fed rats, *Cyanobacteria* were the most differentially abundant taxa (phylum, *p* < 0.01), which were driven by the class *4C0d-2* (*p* < 0.01) and order *YS2* (*p* < 0.01; Figure 3A, Figure S1). Their abundance was significantly reduced by HF feeding (LF vs. HF, *p* < 0.01), and this change was restored to the LF level in HF/Pro and HF/Pca groups (HF vs. HF/Pro, *p* < 0.05; vs. HF/Pca, *p* < 0.05).

Probiotic supplementation led to a particular increase in the HF/Pro group in *Lentisphaerae* (phylum, *p* < 0.01) abundance, which was primarily driven by increased abundance in *Lentisphaeria* (class, *p* < 0.01), *Victivallaceae* (family, *p* < 0.01), and *Victivallales* (genus, *p* < 0.01; Figure 3A–D, Figure S1).

HF/Pca rats exhibited a specific increase in *TM7* (phylum, *p* < 0.05) abundance and this profile was mainly characterized by increased abundance of *TM7-3* (class, *p* < 0.05), *CW040* (order, *p* < 0.05), and *F16* (family, *p* < 0.05, Figure S1).

At the species level (Figure 3F–I), there was no significant difference in *Bifidobacterium* abundance among groups, but the abundance was significantly higher in the HF/Pca group compared to the LF group (*p* < 0.05). *L. paracasei* and *L. zeae* were only present in probiotic-treated groups with a significant difference between HF/Pro and HF/Pca groups (*p* < 0.05 and *p* < 0.01, respectively). The presence of *Prevotella* was detected only in LF and HF/Pca groups without a significant difference between them.

**Figure 4:**
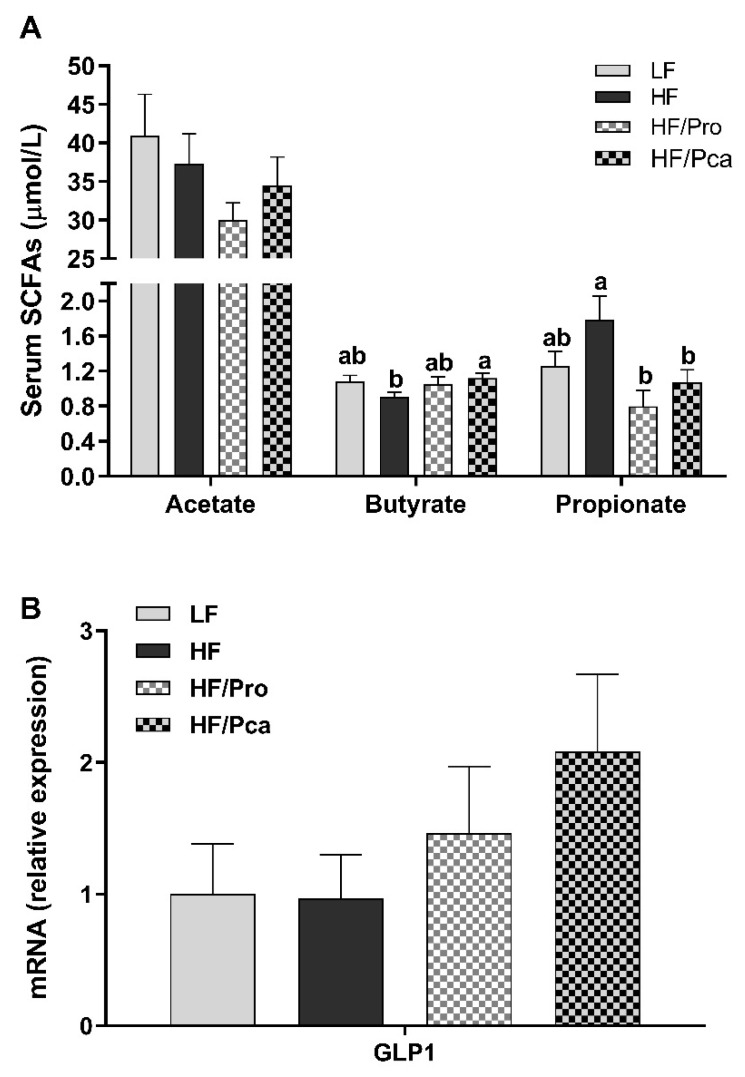
Compositional changes in gut microbiota by non-encapsulated or encapsulated probiotic supplementation were associated with changes in serum concentration of SCFAs (**A**) with no significant difference in gene expression of GLP-1 in the ileum (**B**) of rats fed an LF, HF, HF/Pro, or HF/Pca diet for seven weeks. Values are means ± SEMs; *n* = 8/group. ^a,b,c^ means with different letters are statistically significant, *p* < 0.05. GLP-1, glucagon-like peptide 1; HF, high fat; HF/Pca, HF with supplemented with encapsulated *L. casei* W8 (4 × 10^7^ cfu/g diet); HF/Pro, HF supplemented with non-encapsulated *L. casei* W8 (4 × 10^7^ cfu/g diet); LF, low fat; SCFA, short chain fatty acid.

Probiotic supplementation led to changes in serum concentration of SCFAs (Figure 4A). HF feeding alone led to changes in serum SCFA levels with a non-significant decrease in butyrate and increase in propionate compared to the LF control group. This change was partially and/or significantly reversed by probiotic supplementation (butyrate: HF vs. HF/Pca, *p* < 0.05; propionate: HF vs. HF/Pro, *p* < 0.01, vs. HF/Pca, *p* < 0.05). There was no difference in acetate levels among groups. Correlation analysis showed a significantly positive relationship between serum butyrate and abundance of *Cyanobacteria* (*r* = 0.43; *p* < 0.05) and *Prevotella* (*r* = 0.41; *p* < 0.05). Serum propionate was negatively correlated with *L. paracasei* (*r* = −0.39; *p* < 0.05) and *L. zeae* (*r* = -0.37; *p* < 0.05).

No significant differences were found among groups for GLP gene expression, but the HF/Pca group had the highest GLP-1 mRNA expression (Figure 4B).

### 3.4. Effects of Probiotic Administration on GI Barrier Integrity and Inflammation

**Figure 5:**
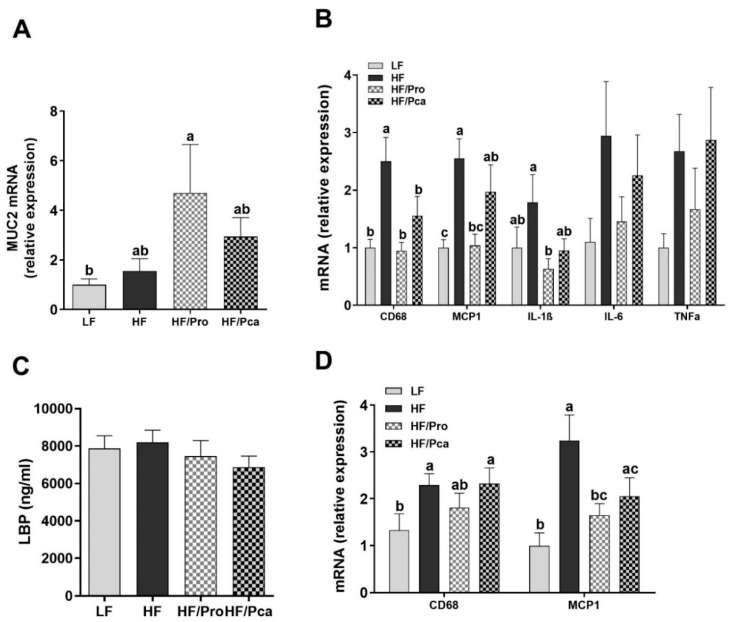
Non-encapsulated probiotic supplementation improved markers of gut integrity and inflammation. Gene expressions of MUC2 (**A**) and inflammatory markers (**B**) in the ileum, serum LBP (**C**), and gene expression of macrophage infiltration markers (**D**) in mesenteric fat of rats fed an LF, HF, HF/Pro, or HF/Pca diet for seven weeks. Values are means ± SEMs; *n* = 8/group. ^a,b,c^ means with different letters are statistically significant, *p* < 0.05. CD68, cluster of differentiation 68; HF, high fat; IL-1β, interleukin-1 beta; IL-6, interleukin-6; LBP, lipopolysaccharides-bind protein; LF, low fat; MCP1, monocyte chemoattrantant protein 1; MUC2, mucin 2; HF/Pca, HF with supplemented with encapsulated *L. casei* W8 (4 × 10^7^ cfu/g diet); HF/Pro, HF supplemented with non-encapsulated *L. casei* W8 (4 × 10^7^ cfu/g diet); TNFα, tumor necrosis factor alpha.

Gene expression of a mucus protein, mucin 2 (MUC2), in the ileum was elevated in the all HF-fed groups, but only the HF/Pro group showed a significant difference compared to the LF group (*p* < 0.05; Figure 5A).

In the distal gut (ileum), HF feeding was associated with significant upregulation in gene expression of macrophage infiltration markers, CD68 (HF vs. LF, *p* < 0.01) and MCP1 (HF vs. LF, *p* < 0.001), which was normalized by probiotic supplementation (HF/Pro) and partially normalized in the encapsulated group (HF/Pca) (CD68, HF vs. HF/Pro, *p* < 0.01; HF vs. HF/Pca, *p* < 0.05; MCP1, HF vs. HF/Pro, *p* < 0.01; HF vs. HF/Pca, ns; Figure 5B). A similar trend was observed in gene expression of inflammatory markers, IL-1β (HF vs. HF/Pro, *p* < 0.05; HF vs. HF/Pca, ns), as well as IL-6 and TNFα, but without significance.

There were no significant differences in circulating LBP (proxy for LPS) levels among groups (Figure 5C).

In mesenteric fat tissue, HF feeding was associated with significant increases in CD68 and MCP1 gene expression (CD68, HF vs. LF, *p* < 0.05; MCP1, *p* < 0.001). MCP1 gene expression was normalized by probiotic supplementation and partially normalized in the encapsulated group (HF vs. HF/Pro, *p* < 0.05; vs. HF/Pca, *p* = 0.07; Figure 5D).

## 4. Discussion

In this current study, we investigated the potential effects of L. casei W8 supplementation on HF diet-induced metabolic changes with or without pectin microencapsulation of the probiotic bacteria for better survivability. Our hypothesis was that seven weeks of probiotic supplementation as a part of HF diet would improve inflammatory state and glucose tolerance in association with compositional changes in gut microbiota and improvement in gut barrier function and that these effects would be enhanced by microencapsulation. HF-driven increases in the ratio of *Firmicutes* to *Bacteroidetes* and *Deltaproteobacteria* abundance and decreases in *Cyanobacteria* abundance were normalized to some extent by non-encapsulated probiotic treatment, but not by the encapsulated probiotic. This data suggests that the probiotic reduced HF-driven dysbiosis and this effect was lost with the encapsulation. Positive changes in microbiota composition in the HF/pro group were associated with a significant upregulation of MUC2 gene expression in the ileum, suggesting improvement of gut barrier integrity. Additionally, non-encapsulated probiotic treatment suppressed local (CD68, MCP1 and IL-1β in the ileum) and systemic (CD68 and MCP1 in visceral adipose fat) inflammation indices, but had no protective effect on glucose tolerance and adiposity. On the other hand, improvements in glucose tolerance and insulin sensitivity were observed only in the encapsulated probiotic-treated group. Interestingly, *Prevotella*, which is associated with improved glucose control, was present only in the HF/Pca group among HF-fed rats in line with the glucose tolerance outcome of this study (Table 1).

In rats, consumption of HF diet (45% fat) for more than five weeks significantly increases bodyweight, energy intake, and fat mass [34]. In this study, HF feeding for seven weeks brought no significant differences in overall bodyweight, and energy intake between LF and HF fed groups. Our control LF group was fed a refined LF as a control diet with identical high sugar level (17% kcal) as the HF diets. In rats, chronic consumption of this specific LF diet has previously been shown to reduce sensitivity to gut satiety peptide cholecystokinin (CCK) [35] and increase intake, bodyweight and adiposity [36] when compared to a chow diet. Thus, it is possible that our diet composition led to a lack of bodyweight difference between the LF and HF groups. However, we still observed an increase in adiposity in HF-fed rats compared to the LF group. Dietary fats rather than bodyweight has previously been shown to regulate adiposity [37].

Unexpectedly, probiotic treatment did not prevent HF-diet driven increase in adiposity and bodyweight, regardless of encapsulation. The non-encapsulated probiotic-treated group exhibited the strongest obesity phenotype compared to the LF group. 

To understand the limited effects of supplementation on the obesity phenotype, we characterized the animals’ microbiota composition. HF consumption resulted in modest changes in gut microbiota composition, which were modulated by probiotic supplementation. At the phylum level, HF feeding led to increased *Firmicutes* abundance and decreased *Bacteroidetes* abundance. Elevated *Firmicutes* to *Bacteroidetes* ratio is a characteristic of obesity-driven disruptions in microbiota, i.e., dysbiosis [4,38]. HF feeding also led to significant increases in *Actinobacteria and Deltaproteobacteria*, which were primarily driven by the elevated abundance of genus *Adlercreutzia* and *Bilophila*, respectively. Studies have demonstrated that an increase in *Adlercreutzia* abundance is associated with intestinal permeability [39], while *Bilophila* blooms with HF diets [40]. The overall effect of HF diet on microbiota composition was modest, and this may be related to the sugar contents of our LF diet, we have previously found that this specific refined diet induces some level of dysbiosis [36].

Unsurprisingly, the probiotic treatments modulated gut microbiota composition. The increased *Firmicutes* to *Bacteroidetes* ratio was notably normalized by the non-encapsulated supplementation and partially improved by the encapsulated treatment. *L. paracasei*, the probiotic species used in this study, was only present in probiotic-treated groups although it was significantly lower in the HF/Pca group. Interestingly, *Lactobacillus* species, notably *Lactobacillus plantarum, Lactobacillus rhamnosus*, and *Lactobacillus paracasei*, appear to be transient passengers rather than to be GI commensals, promoting the growth of indigenous beneficial gut microbes, such as *Bifidobacteria* and leading to ecological rearrangement of gut microbiota [41,42]. In this study, the presence of *L. zeae*, whose abundance has been shown to be significantly increased by *L. casei* BL23 treatment [43], was only detected in probiotic-treated groups, especially the non-encapsulated treated animals. Non-encapsulated probiotic supplementation appears to have a stronger effect on microbiota composition than encapsulated treatment. One possible explanation is that the physical accessibility of encapsulated probiotics may have been limited in our study. The pectin-based coating may have prevented the complete release of encapsulated probiotics into the distal gut, resulting in excretion through feces without exerting its full effects on the host [44]. Low methoxy pectin with added rice bran, protein or other biopolymers have lower porosity [45], and hence, lower release rates. Low-methoxyl pectin-calcium gels form long, rigid junction zones in addition to short crosslinks between chains [46]. The blockwise charge on the pectin used in this study promotes strong gels with high encapsulation rates and high stability [27]. In addition, the particle size of the pectin used was approximately 0.6 µm, compared to 1-4 µm of a typical probiotic cell [44]. It is feasible that pectin could physically cover the entire surface and encapsulate the probiotic cell in a hydrogel matrix, lowering its accessibility. It is, however, important to note that we collected cecal contents for metagenomics analysis and further pectin degradation could have happened in the colon. Since the composition of gut microbiota changes along the GI tract [47,48], despite the positive effect on gut microbiota as discussed previously, the HF/Pro animals showed increased bodyweight and adiposity. Gut microbial-derived SCFAs can modulate the host energy metabolism not only by increasing energy harvest from diets, but also by controlling energy expenditure [49]. Propionate is known to increase energy expenditure through sympathetic activation [50]. In this study, serum propionate was negatively correlated with the abundances of *L. paracasei* and *L. zeae*. whose abundances were elevated in the non-encapsulated treated groups and positively correlated with adiposity, indicating a possibly increased energy efficiency by the probiotic treatment. 

We hypothesized that changes in gut microbiota would be associated with improved gut epithelial integrity and decreases in local and systemic inflammation. HF consumption promotes intestinal inflammation, leading to the altered intestinal barrier function and increases in gut permeability and translocation pro-inflammatory bacterial products, such as LPS, into the circulation [4]. Previous studies have demonstrated that probiotics can improve epithelial barrier integrity by upregulating the expression of mucin from the host [51]. Mucin, as the primary glycoprotein of the GI mucus layer in the GI tract, protects the intestinal epithelium from pathogenic invasion by forming a defensive physicochemical barrier [52]. In this study, we found that probiotic supplementation increased MUC2 gene expression in the ileum. Along with the improvement in gut barrier integrity, we also found that probiotic supplementation normalized HF diet-induced increases in macrophage accumulation (CD68 and MCP1) and inflammation (IL-1β) in the ileum, suggesting a protective effect of the probiotic against local inflammation. Consistently, MCP1 gene expression was suppressed in mesenteric fat of HF/Pro, indicating an anti-inflammatory effect of the probiotic against systemic inflammation.

Inflammatory cytokines can impair glucose tolerance and insulin sensitivity [53]. In response to a glucose challenge, HF fed rats displayed only a modest increase in glycemia. A study by Sumiyoshi et al. demonstrated that chronic intake of high-sucrose diets could induce glucose intolerance [54]. Thus, it is possible that our diet composition high in sugars (17 kcal% in all groups) was responsible for the lack of glycemia difference between LF and HF groups. However, during the OGTT, HF rats showed elevated serum insulin levels, suggesting impaired insulin signaling. Systemic insulin resistance has been associated with adipose tissue inflammation in visceral depots [55]. In the present study, the inflammatory profile of adipose tissue supports the impaired insulin signaling in the HF group.

Surprisingly, the glycemic level was further elevated in the HF/Pro group. The HF/Pro group showed higher adiposity than all other groups. Since excess fat mass, especially visceral, has been linked to glucose metabolism dysregulation [56], high visceral adiposity may have driven hyperglycemia in the HF/Pro group. Insulin levels in the HF/Pro group were partially normalized, suggesting some improvement in insulin-sensitivity. Overall, despite a reduction in inflammatory status in the non-encapsulated probiotic group, supplementation had limited effects on glucose tolerance, suggesting that chronic inflammation is one of several factors involved in obesity-associated alteration in glucose homeostasis.

It is, however, important to point out that different metabolic outcomes were observed between HF/Pro and HF/Pca groups for inflammatory profiles, and glucose tolerance. While both probiotic-treated groups showed improvements in local and systemic inflammation and gut barrier functions against the HF challenge, the effects were only significant in rats receiving non-encapsulated probiotics, suggesting the encapsulation process did not enhance the probiotic effects. These results cast doubt on the efficacy of encapsulation employed in this study above the efficacy of probiotic addition as discussed previously.

Conversely, during the OGTT, the HF-induced glucose intolerance was further deteriorated in the HF/Pro group while the impairment was alleviated in the HF/Pca group. This suggests a positive effect of encapsulation on glucose tolerance, potentially independent of the probiotics. As discussed previously, pectin was used for encapsulation. Even allowing for its minimal concentration in diets, it is feasible that pectin itself exerted prebiotic and/or synbiotic effects via hydrolysis and oligosaccharides fermentation Selective fermentation of the branched, neutral sugar rich domain of pectin may have generated favorable metabolites [57], such as SCFAs. Even if the hydrogel stayed intact, the metabolites from pectin fermentation are small and could have diffused from the pectin gel without the probiotic bacteria being released. Studies have shown an increase in butyrate concentration after feeding animals with pectin-containing diets [58,59]. Dietary supplementation of butyrate can improve insulin sensitivity and glucose tolerance [60]. Interestingly, in this study, the HF-induced decrease in serum butyrate was restored in HF/Pca rats. SCFA production is associated with increased abundance and diversity of gut microbiota [61]. In this study, there was a positive correlation between serum butyrate and *Prevotella* abundance, which characterized the microbial profile of the HF/Pca group. *Prevotella* abundance increases with carbohydrate-rich diets, especially fibers [62] and is associated with improved glucose control [63]. Considering that fiber contents were controlled for this study, the increase in *Prevotella* abundance in the HF/Pca group may have been due to the presence of pectin.

Moreover, as one of the most commonly used probiotics, *Bifidobacterium* abundance was highest in the HF/Pca group, which was significantly higher than the LF group. *Bifidobacterium* has been found to be associated with improved glucose tolerance by elevating the level of GLP-1, a known incretin [15], in both gut and plasma in mice fed an HF diet [64], which corresponds to glucose tolerance outcome of this study. It is also interesting to note that *Bifidobacterium* abundance was positively correlated with the gene expression of GLP-1 in the ileum in this study (*r* = 0.56; *p* < 0.01). 

## 5. Conclusions

In conclusion, we demonstrated that probiotic-induced changes in gut microbiota composition were associated with improvements in gut barrier functions and local and systemic inflammation in HF-fed rats. Encapsulation of the probiotics with pectin in this study did not enhance the effect of probiotic supplementation. However, pectin encapsulation had positive effects on glucose tolerance, possibly due to prebiotic and/or synbiotic effect of pectin used as encapsulation material. Thus, our findings support the potential of using an oral non-encapsulated or encapsulated probiotic supplementation to ameliorate obesity-associated metabolic abnormalities.

## Figures and Tables

**Table 1:** Summary of the changes observed in HF, HF/Pro and HF/Pca rats in comparison to the LF control group.

**Metabolic outcomes (vs. LF)**	**HF**	**HF/Pro**	**HF/Pca**
Body weight	↑	↑*	↑
Adiposity	↑	↑*	↑*
Energy Intake	↑	↑*	↑
Glycemia (OGTT)	↑	↑*	LF*
Insulinemia (OGTT)	↑*	↑	↑
**Microbiota**	**HF**	**HF/Pro**	**HF/Pca**
*Bifidobacterium*			↑*
*L. paracasei*		↑*	
*L. zeae*		↑*	
*Prevotella*	↓	↓	LF*
Butyrate	↓*	LF*	LF*
Propionate	↑*	LF*	LF*
**GI gene expression**	**HF**	**HF/Pro**	**HF/Pca**
GLP-1	-	↑	↑
MUC2	↑	↑*	↑
**Inflammatory profile**	**HF**	**HF/Pro**	**HF/Pca**
GI- CD68	↑*	LF*	LF*
GI- MCP1	↑*	LF*	↑*
GI- IL-1β	↑	↓	-
Mes Fat - CD68	↑*	LF*	↑*
Mes Fat - MCP1	↑*	LF*	↑*

↑ and ↓ indicate increases and decreases, respectively; asterisk shows a statistical significance. LF indicates normalization to LF levels. CD68, cluster of differentiation 68; GI, gastrointestinal; GLP-1, glucagon-like peptide-1; HF, high fat; HF/Pca, HF with supplemented with encapsulated L. casei W8 (4 × 10^7^ cfu/g diet); HF/Pro, HF supplemented with non-encapsulated L. casei W8 (4 × 10^7^ cfu/g diet); IL-1β, interleukin-1 beta; LF, low fat; MCP1, monocyte chemotactic protein 1; MUC2, mucin 2; OGTT, oral glucose tolerance test.

## Supplementary Materials

The following are available online at https://www.mdpi.com/2072-6643/11/9/1975/s1, Table S1: Diet Composition of LF, HF, HF/Pro and HF/Pca diets, Table S2: Macronutrient composition of LF, HF, and HF_BB diets as a percent of energy, Table S3: Primer sequences used for qPCR, Figure S1: Cladogram generated from LDA score of 2.5 shows the most differentially abundant taxa enriched in microbiota from rats fed an LF, HF, HF/Pro, or HF/Pca diet for seven weeks.
