# Supplementary material for: Beneficial Effects of Non-Encapsulated or Encapsulated Probiotic Supplementation on Microbiota Composition, Intestinal Barrier Functions, Inflammatory Profiles, and Glucose Tolerance in High Fat Fed Rats
Source: Nutrients. 2019 Aug 22;11(9):1975. doi: 10.3390/nu11091975 (PMC6769526; doi:10.3390/nu11091975)
Supplement: Supplementary file 1 [file nutrients-11-01975-s001.pdf]

Table S1. Diet Composition of LF, HF, HF/Pro and HF/Pca diets[1]

Diet Ingredient	LF - D12450H	HF - D12451	HF/Pro - D12451	HF/Pca - D12451
			g	
Casein, 30 Mesh	200	200	200	200
L-Cystine	3	3	3	3
Corn Starch	452.2	72.8	72.8	72.8
Maltodextrin 10	75	100	100	100
Sucrose	172.8	172.8	172.8	172.8
Cellulose, BW200	50	50	50	50
Soybean Oil	25	25	25	25
Lard	20	177.5	177.5	177.5
Mineral Mix S10026	10	10	10	10
Dicalcium Phosphate	13	13	13	13
Calcium Carbonate	5.5	5.5	5.5	5.5
Potassium Citrate, 1 H20	16.5	16.5	16.5	16.5
Vitamin Mix V10001	10	10	10	10
Choline Bitartrate	2	2	2	2
FD&C Yellow Dye #5	0.04	0	0	0
FD&C Red Dye #40	0.01	0.05	0.05	0.05
Intact L. casei W8			0.0076	
Microencapsulated L. casei W8				8.58
Total	1055.03	858.15	858.1576	866.7315

[1]*Lactobacillus paracasei subsp. paracasei* L. casei W8® (L. casei W8) was microencapsulated with pectin beads, consisting of low methyl pectin, water, $CaCl_2$, and L. casei w8. HF, high fat; LF, low fat; HF, high fat; HF/Pca, HF with supplemented with encapsulated *L. casei* W8 ($4x10^7$ cfu /g diet); HF/Pro, HF supplemented with non-encapsulated *L. casei* W8 ($4x10^7$ cfu/g diet).

Table S2. Macronutrient composition of LF, HF, and HF_BB diets as a percent of energy[1]

Diet Ingredient	LF - D12450H	HF - D12451	HF/Pro - D12451	HF/Pca - D12451
	% Kcal			
Protein	20	20	20	20
Carbohydrate	10	35	35	35
Sucrose	17	17	17	17
Fat	70	45	45	45
**Total**	100	100	100	100
	Kcal/g diet			
	3.82	4.7	4.7	4.7

[1] Diets prepared by Research Diets, Inc. HF, high fat; LF, low fat; HF, high fat; HF/Pca, HF with supplemented with encapsulated *L. casei* W8 ($4x10^7$ cfu /g diet); HF/Pro, HF supplemented with non-encapsulated *L. casei* W8 ($4x10^7$ cfu/g diet).

Table S3. Primer sequences used for qPCR[1]

Gene*	Forward primers (5' to 3')	Reverse primers (5' to 3')	Accession number
**β-actin**	ACGGTCAGGTCATCACTATC	GATGCCACAGGATTCCATAC	NM_031144.3
**CD68**	AAACAGTCCAGGCTTCTC	ATGGCTGGGAACCATTAG	NM_001031638.1
**GAPDH**	GAGCATCTCCCTCACAATTC	GGGTGCAGCGAACTTTAT	NM_017008.4
**GLP1**	CCAAGCAAGGAGAGAGAAAC	GATGACCAAGGCAGAGAAAG	NM_012728.1
**IL-1β**	CATTGTGGCTGTGGAGAA	GCAGTGCAGCTGTCTAAT	NM_031512.2
**IL-6**	TGTTGTGGGTGGTATCCT	CCTTCTTGGGACTGATGTTG	NM_012589.2
**MCP1**	GGCTGGAGAACTACAAGAG	TCTGGACCCATTCCTTATTG	NM_031530.1
**MUC2**	CTGAGGAAGGCCAAGTTTAC	CAGGTCCCAGAGAGGAAATA	U68172
**TNFα**	TCTACTCCCAGGTTCTCTTC	GCTGACTTTCTCCTGGTATG	NM_012675.3

[1]Cd68, cluster of differentiation 68; GAPDH, glyceraldehyde 3-phosphate dehydrogenase; GLP1, glucagon-like peptide-1; GPR41, -43, G protein-coupled receptor 41, -43; IL-1β, interleukin-1 beta; IL-6, interleukin-6; MCP1, monocyte chemotactic protein 1; MUC2, mucin 2; TNFα, tumor necrosis factor alpha.

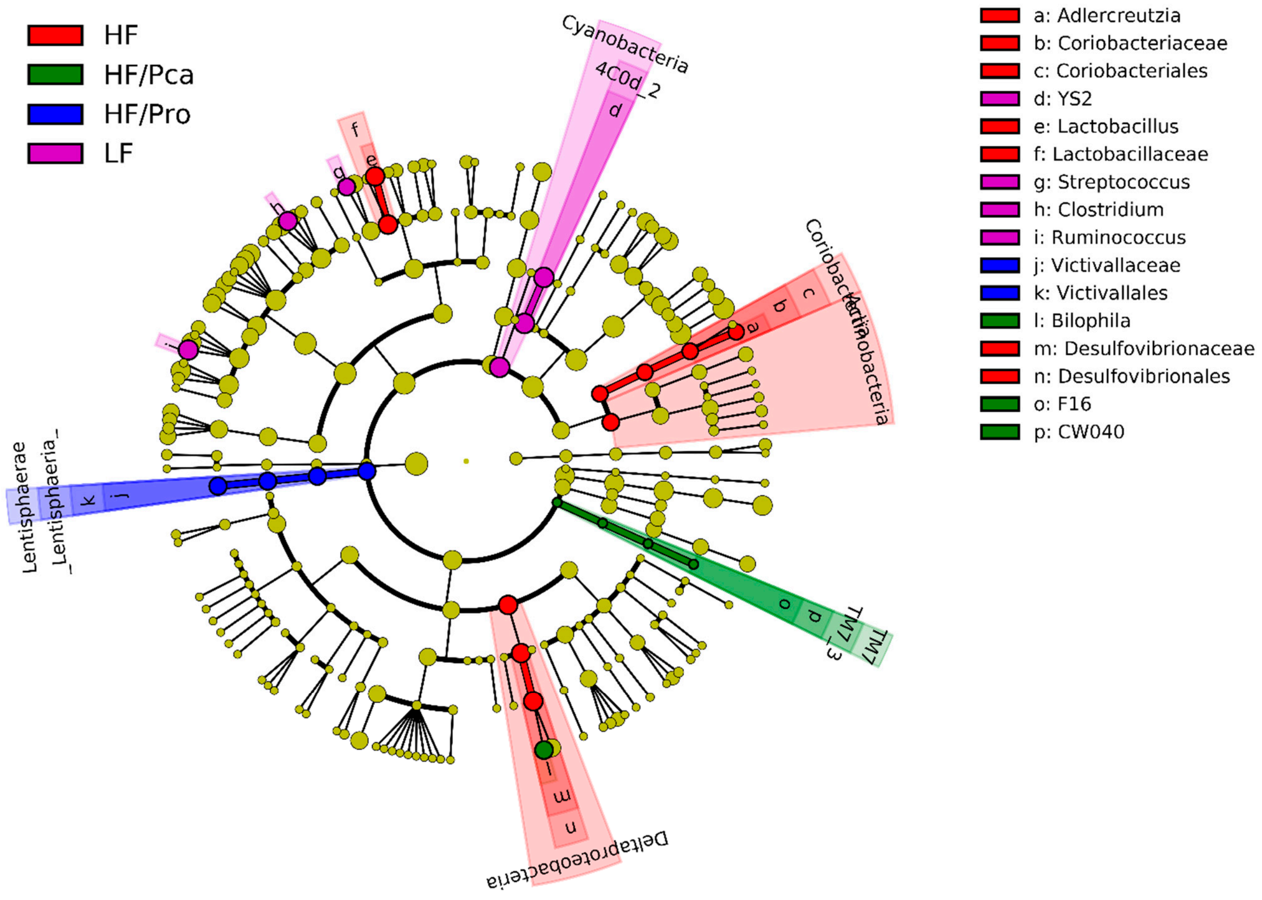

Figure S1. Cladogram generated from LDA score of 2.5 shows the most differentially abundant taxa enriched in microbiota from rats fed an LF, HF, HF/Pro, or HF/Pca diet for 7 weeks (green indicating LF, red HF, magenta HF/Pro, blue HF/Pca, and yellow indicating non-significance). n = 8/group. The six rings of the cladogram stand for domain (innermost), phylum, class, order, family and genus. LEfSe analysis was performed on log-transformed abundances using the Galaxy online module to identify discriminant taxa among groups. HF, high fat; LF, low fat; LEfSe, linear discriminant analysis (LDA) effect size; HF/Pca, HF with supplemented with encapsulated *L. casei* W8 ($4x10^7$ cfu /g diet); HF/Pro, HF supplemented with non-encapsulated *L. casei* W8 ($4x10^7$ cfu/g diet).
